# Clinical characteristics and pregnancy outcomes of atypical hemolysis, elevated liver enzymes, and low platelets syndrome: A case series

**DOI:** 10.1097/MD.0000000000019798

**Published:** 2020-05-01

**Authors:** Ruoan Jiang, Ting Wang, Baohua Li, Jing He

**Affiliations:** Department of Obstetrics and Gynecology, Women's Hospital, Zhejiang University of Medicine, Hangzhou, Zhejiang Province, China.

**Keywords:** hemolysis, elevated liver enzymes, and low platelets syndrome, hypertension, maternal complications, preeclampsia, pregnancy

## Abstract

**Rationale:**

Hemolysis, elevated liver enzymes, and low platelets (HELLP) syndrome is a serious and rare disease, which is secondary to preeclampsia in most cases. Hypertension is usually considered as a premonitory symptom of HELLP syndrome. In some patients with HELLP syndrome; however, they develop hypertension very late, even after liver enzymes are elevated or platelet count is decreased. This condition is known as atypical HELLP syndrome.

**Patient concerns:**

We screened and identified 4 cases of atypical HELLP syndrome in our hospital database from January 2007 to December 2018. All patients had a history of nonspecific symptoms for a few days before hospital admission, such as dizziness, nausea, and vomiting. They developed hypertension after abnormalities were noted in liver enzymes and platelet count.

**Diagnoses:**

They were diagnosed with atypical HELLP syndrome.

**Interventions:**

These patients received same treatments as those with HELLP syndrome. Two patients took oral antihypertensive treatment to normalize the blood pressure.

**Outcomes:**

In our patients, both mothers and neonates had favorable outcomes. In follow-ups, they reported no incidences of high blood pressure after recovery from atypical HELLP syndrome.

**Lessons:**

These cases provided additional clinical evidences of atypical HELLP syndrome. The incidence of atypical HELLP syndrome is extremely low. Hypertension is not essential for the diagnosis of HELLP syndrome, and can even appear after the onset of laboratory abnormalities. Advanced age, multiple pregnancies, hepatitis B virus infection, and obesity may be potential risk factors for atypical HELLP syndrome. Blood pressure should be monitored closely after delivery.

## 1 Introduction

Hemolysis, elevated liver enzymes, and low platelets (HELLP) syndrome has been recognized as a serious complication of preeclampsia and eclampsia for many years.^[1]^ Sibai reported that although a diagnosis of HELLP syndrome is finally made, hypertension and/or proteinuria may be absent in 10% to 15% of patients.^[2]^ Martin also reported that 5% to 6% patients of HELLP syndrome with platelet (PLT) nadir <100 × 10^9^ cells/L had no elevated diastolic blood pressures (≥90 mm Hg), and 2% to 4% never exhibited systolic hypertension.^[3]^ Therefore, Sibai proposed that cases presenting with signs and symptoms of preeclampsia and either hemolysis or elevated liver enzymes or low PLTs, but with no hypertension or proteinuria, should be classified as atypical HELLP syndrome.^[4]^

Between January 2007 and December 2018, a total of 525 cases of HELLP syndrome were identified among the records of 175,338 pregnant women (0.3%) in the database of Women's Hospital, Zhejiang University. Among them, we found 4 patients with abnormal laboratory test results (elevated liver enzymes or low PLTs) at onset and subsequently developed hypertension. These patients were diagnosed with atypical HELLP syndrome. Atypical HELLP syndrome is not easy to diagnose in the early stage, and there are few studies concerning this disease entity.

## 2 Case presentation

### 2.1 Case 1

A 37-year-old, gravid 1, para 0 (G1P0) woman with twin pregnancy was admitted at 30 weeks of gestation due to nausea and vomiting. She had advanced maternal age, hepatitis B virus (HBV) infection, and gestational diabetes mellitus. Her 24-hour urinary protein level was 410 mg. However, her blood pressure was normal (115/62 mm Hg). Blood tests indicated a PLT count of 96 × 10^9^/L, alanine aminotransferase (ALT) level of 133 U/L, and aspartate aminotransferase (AST) level of 116 U/L. Compound glycyrrhizin intravenously injection 60 mL was administered to reduce the liver enzymes once per day before delivery. A review of her medical records showed that liver enzymes, PLT count, and blood pressure were all normal before admission. Cesarean section was performed at 30 3/7 weeks. The newborn infants had birth weights of 1050 g and 1000 g, and Apgar scores of 9 to 10 and 6 to 8 (1–5 min), respectively. Repeated high blood pressure was detected on postpartum day 2 (up to 160/92 mm Hg) and antihypertensive treatment with oral labetalol was performed. The PLT decreased to 77 × 10^9^/L at the lowest, whereas ALT and AST increased to 239 U/L and 153 U/L at the highest, respectively, after cesarean delivery. Without specific treatment, PLT count returned spontaneously to the normal reference range 3 days after cesarean section. Reduced glutathione sodium 1.2 g and compound glycyrrhizin 60 mL were intravenously prescribed to improve liver functions once per day for 1 week. Her blood pressure decreased to normal after 14 days. The patient underwent another surgery due to fat liquefaction of abdominal incision on postpartum day 12.

### 2.2 Case 2

A 35-year-old, G2P1 pregnant woman was admitted at 37 4/7 weeks of gestation due to epigastric pain. She had advanced maternal age, HBV infection (HBV DNA <500 copies), and a history of cesarean section due to severe preeclampsia at 28 weeks of gestation. Her blood pressure recordings and laboratory tests were all normal at previous routine pregnancy check-ups. Blood test after admission showed a PLT count of 96 × 10^9^/L, with ALT of 75 U/L, and AST of 54 U/L. Delivery by cesarean section was performed because of regular contractions. The newborn infant had a birth weight of 3100 g, and Apgar scores of 10 to 10 (1–5 min). The blood pressure of the woman was 137/73 mm Hg with positive urinary protein (++) before cesarean section. High blood pressure was detected at 24 hours postpartum, up to 157/82 mm Hg on postpartum day 2. Without specific treatment, hepatic enzymes, and PLT count returned spontaneously to the normal reference range, and blood pressure decreased to normal 2 days after cesarean section.

### 2.3 Case 3

A 41-year-old, G8P1 pregnant woman was admitted at 35 4/7 weeks of gestation due to premature membrane rupture. She had advanced maternal age and HBV infection (HBV DNA <500 copies). Emergency cesarean section was performed due to premature membrane rupture, breech presentation, and regular contractions. The newborn infant had a birth weight of 3050 g, and the Apgar score was 6 to 8 (1–5 min). This woman had normal blood pressure recordings before admission. On postpartum day 1, she complained of dizziness, and her blood pressure was 130/71 mm Hg with positive urinary protein excretion. Postoperative blood test showed a PLT count of 96 × 10^9^/L, with ALT of 48 U/L, and AST of 54 U/L. High blood pressure were detected on postpartum day 5, reaching 160/100 mm Hg on postpartum day 6, and pleural effusion developed. Antihypertensive treatment with oral labetalol was performed and blood pressure returned to normal on postoperative day 18. Without specific management, the laboratory profiles spontaneously returned to normal 4 days after cesarean section.

### 2.4 Case 4

A 30-year-old, G2P0 woman with triple pregnancy was admitted at 33 0/7 weeks of gestation due to dizziness lasting for 6 days. She also had intrahepatic cholestasis of pregnancy. Her blood pressure was 128/89 mm Hg and laboratory examinations were all normal before admission. On admission, urinary protein was negative on routine urine test. Blood tests; however, showed a PLT count of 84 × 10^9^/L, with ALT of 203 U/L, and AST of 189 U/L. Delivery by cesarean section was performed. The newborn infants had birth weights of 2100, 2100, and 1790 g, with Apgar scores of 10–10, 10–8, and 10–8 (1–5 min), respectively. Blood pressure increased markedly during the operation. The patient's high blood pressure lasted for 2 days, and then returned to normal without antihypertensive treatment. Without specific treatment, PLT count returned spontaneously to the normal reference range 2 days after cesarean section. Compound glycyrrhizin injection at a daily dose of 60 mL was intravenously infused once per day for 4 days and hepatic enzymes returned normal 5 days after cesarean section.

All these 4 patients were diagnosed with atypical HELLP syndrome. The clinical characteristics of the 4 patients are summarized in Table 1, and their blood pressure records are listed in Table 2. Laboratory profiles of different stages are listed in Table 3. These patients received the same treatments as patients with HELLP syndrome. Telephone follow-up was performed, and all patients were confirmed to have had no incidences of high blood pressure after recovery from atypical HELLP syndrome. The follow-up periods ranged from 43 to 146 months.

**Table 1:**
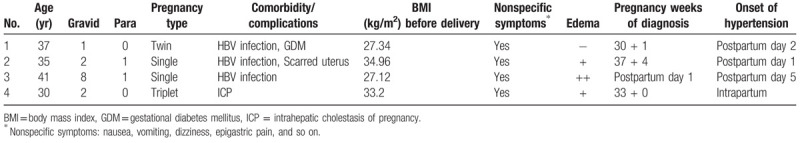
Clinical characteristics of 4 pregnant women with atypical HELLP syndrome.

**Table 2:**
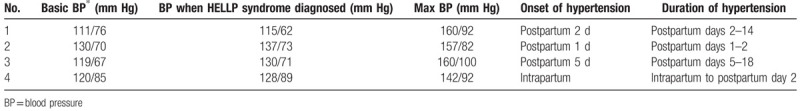
Blood pressure records of 4 pregnant women with atypical HELLP syndrome.

**Table 3:**
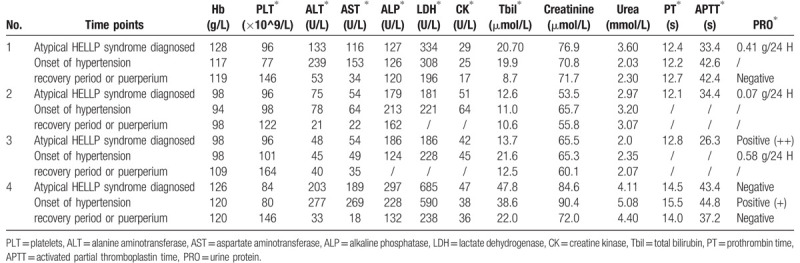
Laboratory profiles of 4 pregnant women with atypical HELLP syndrome.

This report was approved by the Institutional Review Board, Women's Hospital, School of Medicine, Zhejiang University (Approval number: 20170161). All patients have provided informed consent for publication of the case details.

## 3 Discussion

In this case study, we presented 4 cases of atypical HELLP syndrome, whose elevated liver enzymes and low PLTs occurred early than hypertension. In all patients, hypertension occurred postpartum, which further confirmed the diagnosis of HELLP syndrome.

HELLP syndrome is a serious condition that may result in a series of adverse maternal and neonatal consequences.^[5,6]^ Up till now, HELLP syndrome is considered as one of the most serious forms of preeclampsia, not as a separate disorder. Low PLTs may result from increased PLT activation, aggregation, and consumption and is regarded as a marker of disease severity. Significantly impaired liver functions, such as elevated blood concentrations of liver transaminases, may be observed in women with severe preeclampsia.^[7,8]^ In liver dysfunction, AST is the dominant transaminase released into the peripheral circulation and is associated with periportal necrosis.^[8]^

HELLP occurs in 0.2% to 0.9% of pregnancies, and it occurs secondary to preeclampsia in 70% to 80% of cases. Hypertension is present in most HELLP cases, but signs of preeclampsia may be subtle or missing.^[2,9]^ Sibai summarized 5 articles and found that hypertension was absent in 12% to 18% of a total of 1117 cases of HELLP syndrome^[2]^; this condition was referred as atypical HELLP syndrome. Regardless of the presence or absence of hypertension or proteinuria, therapeutic principle for atypical HELLP syndrome is similar to classical HELLP syndrome. Over the past 10 years, there are a number of reports to clarify the diagnosis, pathogenesis, risk factors, and management of this disease, but little progress has been made. In addition, few groups have reported the incidence and clinical features of atypical HELLP syndrome.

Compared to women without HELLP syndrome, women with HELLP syndrome are more likely to be >35 years old (22% vs 33%, respectively), nulliparous (43% vs 67%, respectively), to have had a previous gestational hypertensive disorder (7% vs 9%, respectively), and to have experienced multiple pregnancy (2% vs 7%, respectively).^[10]^ However, high quality randomized trials are absent to identify risk factors of HELLP syndrome, especially for atypical HELLP.

In our study, 4 cases of atypical HELLP were identified among 525 cases of HELLP syndrome in our hospital. Among them, 2 cases (50.0%) were multifetal pregnancies, 3 cases (75.0%) had advanced maternal age, and the average body mass index was 30.655 kg/m^2^. Due to the limited sample size, we could not draw conclusions about the risk factors for atypical HELLP syndrome. However, taking other researches into consideration, advanced maternal age, multifetal pregnancy, HBV infection, and obesity are potential risk factors of atypical HELLP syndrome. Sibai^[11]^ and Tomsen^[12]^ proposed that right upper quadrant pain and generalized malaise in up to 90% of HELLP syndrome cases and nausea and vomiting in 50% of cases, which were emphasized and interpreted in the 2019 ACOG Practice Bulletin.^[7]^ All patients had a history of nonspecific symptoms for a few days before hospital admission, such as dizziness, nausea and vomiting, and hypertension occurred within 1 to 5 days after abnormalities in laboratory indicators (hepatic enzymes, PLT counts) in all 4 patients. Therefore, we recommend performing laboratory investigations (complete blood count and liver enzymes) in all pregnant women who have high risk factors with suspected preeclampsia when they present with nonspecific symptoms during the third trimester. These findings indicate that clinicians should be alert when encountering pregnant women with these risk factors, even in the absence of hypertension.

For the 4 cases documented, 3 were diagnosed with atypical HELLP syndrome before delivery. Another patient received emergency cesarean section due to premature membrane rupture and regular contractions. After delivery, abnormal PLT and liver enzymes levels were detected, and the patient was also diagnosed with atypical HELLP syndrome. Interestingly, all these 4 patients developed postpartum hypertension within 5 days after delivery, which helps to confirm the diagnosis of HELLP syndrome. However, no previously published literature has ever reported this condition. Therefore, it seems rational to speculate that the last-onset of hypertension in patients with atypical HELLP syndrome could be a separate entity of this rare syndrome. In our patient, close monitoring after cesarean section, such as blood pressure and pulse, made it possible to timely detect postpartum hypertension. In addition, the blood pressure of patients with potential gestational hypertensive disease may be unstabilized and undetected before delivery. Therefore, patients with atypical HELLP syndrome should receive close perinatal monitoring of vital signs, particularly blood pressure, along with fluid intake and output.

In our patients, both mothers and neonates had favorable outcomes, and during follow-up, all mothers showed normal blood pressures after recovery from atypical HELLP syndrome. Good maternal and neonatal outcomes were associated with early detection and accurate diagnosis, which are essential for management.

In conclusion, this case study reconfirmed the existence of atypical HELLP syndrome in 4 patients, all of whom were normotensive before laboratory tests (hepatic enzyme, PLT count) became abnormal. These cases were characterized by elevated liver enzymes, decreased PLT levels, and lagging hypertension. Some nonspecific symptoms were observed in the early stages of the disease. Advanced age, multiple pregnancy, HBV infection, and obesity were potential risk factors. Pregnant women with these risk factors should be observed closely to diagnose the occurrence of atypical HELLP timely, and blood pressure monitoring after delivery is also necessary.

## Author contributions

**Conceptualization:** Ruoan Jiang.

**Data curation:** Ting Wang.

**Investigation:** Ting Wang.

**Methodology:** Baohua Li.

**Supervision:** Baohua Li, Jing He.

**Writing – original draft:** Ruoan Jiang.

**Writing – review and editing:** Ruoan Jiang, Jing He.

## References

[1] American College of Obstetricians and Gynecologists; Task Force on Hypertension in Pregnancy. Report of the American College of Obstetricians and Gynecologists’ Task Force on hypertension in pregnancy. ObstetGynecol 2013;122:1122–31. 10.1097/01.AOG.0000437382.03963.88 24150027

[2] Sibai BM Diagnosis, controversies, and management of the syndrome of hemolysis, elevated liver enzymes, and low platelet count. Obstet Gynecol 2004;103:981–91. 15121574 10.1097/01.AOG.0000126245.35811.2a

[3] Martin JN Jr Rinehart BK May WL The spectrum of severe preeclampsia: comparative analysis by HELLP syndrome classification. Am J Obstet Gynecol 1999;180:1373–84. 10368474 10.1016/s0002-9378(99)70022-0

[4] Stella CL Sibai BM Preeclampsia: diagnosis and management of the atypical presentation. J Matern Fetal Med 2006;19:381–6. 10.1080/14767050600678337 16923692

[5] Bhattacharya S Campbell DM The incidence of severe complications of preeclampsia. Hypertens Pregnancy 2005;24:181–90. 16036402 10.1081/PRG-200059873

[6] Barton JR Sibai BM Diagnosis and management of hemolysis, elevated liver enzymes, and low platelets syndrome. Clin Perinatol 2004;31:807–33. 15519429 10.1016/j.clp.2004.06.008

[7] ACOG Practice bulletin, no. 202: gestational hypertension and preeclampsia. Obstet Gynecol 2019;133:e1–25. 30575675 10.1097/AOG.0000000000003018

[8] Burrows RF Kelton JG Thrombocytopenia at delivery: a prospective survey of 6715 deliveries. Am J Obstet Gynecol 1990;162:731–4. 2316579 10.1016/0002-9378(90)90996-k

[9] Schaarschmidt W Rana S Stepan H The course of angiogenic factors in early- vs. late-onset preeclampsia and HELLP syndrome. J Perinat Med 2013;41:511–6. 23612628 10.1515/jpm-2012-0248 PMC3928646

[10] Fitzpatrick KE Hinshaw K Kurinczuk JJ Risk factors, management, and outcomes of hemolysis, elevated liver enzymes, and low platelets syndrome and elevated liver enzymes, low platelets syndrome. Obstet Gynecol 2014;123:618–27. 24499757 10.1097/AOG.0000000000000140

[11] Sibai BM The HELLP syndrome (hemolysis, elevated liver enzymes, and low platelets): much ado about nothing? Am J Obstet Gynecol 1990;162:311–6. 2309811 10.1016/0002-9378(90)90376-i

[12] Tomsen TR HELLP syndrome (hemolysis, elevated liver enzymes, and low platelets) presenting as generalized malaise. Am J Obstet Gynecol 1995;172:1876–8. 7778647 10.1016/0002-9378(95)91426-9

